# CompoundDenseNet: a novel approach for accurate recognition of Bangla handwritten compound characters

**DOI:** 10.3389/frai.2026.1751148

**Published:** 2026-04-15

**Authors:** Nazia Alfaz, Talha Bin Sarwar, Fahmid Al Farid, Md Saef Ullah Miah, Sadia Afrin, Shakila Rahman, Jia Uddin, Hezerul bin Abdul Karim

**Affiliations:** 1 Department of Computer Science, American International University–Bangladesh (AIUB), Dhaka, Bangladesh; 2 Faculty of Computer Science and Informatics Berlin School of Business and Innovation, Berlin, Germany; 3 Centre for Image and Vision Computing (CIVC), Centre of Excellence for Artificial Intelligence, Faculty of Artificial Intelligence and Engineering (FAIE), Multimedia University, Cyberjaya, Selangor, Malaysia; 4 Department of AI and Big Data, Endicott College, Woosong University, Daejeon, Republic of Korea

**Keywords:** Adaptive Gradient Algorithm (Adagrad), Adaptive Moment Estimation (Adam), Bangla handwritten compound character, BanglaLekha-Isolated, CMATERdb, Ekush, Dense Convolutional Neural Network (DenseNet), Optical Character Recognition (OCR)

## Abstract

Bangla, one of the most widely spoken languages in the world, presents major challenges in handwritten character recognition because of its complex compound characters with intricate shapes, diverse writing styles, and structural similarities. These features make Bangla a representative example of complex scripts that remain difficult for conventional Optical Character Recognition (OCR) systems. This study focuses on improving the recognition of Bangla handwritten compound characters using a modified DenseNet architecture named CompoundDenseNet. The architecture enhances feature extraction and reuse to better capture the visual variations and fine structural details that existing models often struggle to handle. Its performance was evaluated on three benchmark datasets, BanglaLekha Isolated, Ekush, and CMATERdb, achieving recognition accuracies of 98.5%, 98%, and 96.2% respectively, surpassing previously reported methods. Misclassification analysis using a confusion matrix revealed that the Adam optimizer produced the most stable and accurate results with faster convergence compared to other optimizers tested. While the results demonstrate significant progress, the study also highlights the need for larger and more diverse datasets. Overall, CompoundDenseNet contributes to advancing Bangla handwritten compound character recognition and has the potential to enhance real-world applications such as education, legal documentation, and digital accessibility in Bangla language technologies.

## 1 Introduction

Handwritten character recognition, a fundamental aspect of pattern recognition and artificial intelligence, plays a crucial role in various domains, including commercial and academic OCR, banking, and information retrieval systems (Misgar et al., 2023). OCR technology, specifically for handwritten text, transforms visual data into machine-readable text by analyzing and encoding document characters for data processing. Recent advances in OCR for handwritten manuscripts highlight its growing importance, especially in languages with diverse and complex scripts (Chaudhury et al., 2022; Kumar et al., 2021). Although significant progress has been made for widely used scripts such as Roman (English and European languages) and Asian scripts (Japanese, Korean, and Chinese), many other languages remain less explored.

Bangla, the fifth most spoken language globally, belongs to the Indo-Aryan language family and is the mother tongue of more than 228 million native speakers, with an additional 37 million speaking it as a second language (Samin et al., 2021). It holds official status in Bangladesh and Indian states such as West Bengal, Tripura, and Assam (Rumnaz Imam, 2005). As one of the languages spoken worldwide, Bangla is increasingly gaining attention for its potential applications in OCR, including automatic character evaluation, bank check processing, ID card verification, and postal automation (Das et al., 2010; Basu et al., 2012).

The unique complexity of the Bangla script arises from its alphabet, comprising 50 basic characters (11 vowels and 39 consonants), alongside 334 compound characters formed by combining these basic elements (Reza et al., 2019). Compound characters dominate the Bangla text, accounting for approximately 85% of all characters (Das et al., 2014). These characters exhibit intricate shapes, structural similarities, and significant variability in handwritten forms, presenting unique challenges for OCR systems. Recognizing these characters accurately is further complicated by diverse handwriting styles, morphological complexity, and high visual similarity between certain characters (Roy et al., 2017; Das et al., 2010).

While substantial work has focused on recognizing basic Bangla characters and numerals, The recognition of compound characters remains underexplored. Compound characters often exhibit higher structural complexity, which leads to greater challenges in achieving classification accuracy (Chatterjee et al., 2019). Despite advancements in machine learning and deep learning techniques, existing methods fall short in addressing the nuances of compound characters. For instance, Support Vector Machines (SVMs), though widely used for classifying Bangla characters, struggle with the high-dimensional classification required for nearly 400 distinct classes (Ahsan et al., 2022; Kibria et al., 2020; Das et al., 2010). Deep learning, particularly Convolutional Neural Networks (CNNs), has emerged as a transformative approach to handwritten character recognition, providing state-of-the-art results in fields such as medical imaging, speech recognition, and object detection (Basu et al., 2012; Krizhevsky et al., 2017; Lee et al., 2009). Recent studies utilizing CNN architectures have demonstrated promising results in recognizing Bangla characters. However, their application to compound characters remains limited (Rahman et al., 2015; Shopon et al., 2016; Chatterjee et al., 2019; Basri et al., 2020).

The three main reasons why classification accuracy is lower for compound characters compared to numerals and basic characters are:

- Complex Compound Characters: The Bangla script contains a large number of morphologically complicated characters, often referred to as compound characters. These characters are formed by combining the basic elements or “conjuncts,” as shown in Table 1. Recognizing compound characters accurately is challenging because they can have intricate shapes and structures. OCR systems need to account for the various ways in which these compounds can be formed and written, adding complexity to the recognition process.
- Variability in Handwriting Styles: Different individuals have unique writing styles, resulting in variations in the size, shape, and curvature of characters, even when writing the same script. OCR systems must be robust enough to handle this variability and adapt to different writing styles. It may require a more extensive training dataset to cover the diversity of handwriting styles.
- Structural Similarities: The Bangla script has characters that exhibit structural similarities, making it difficult for OCR systems to distinguish between them accurately. Characters with similar shapes or components can be easily confused, leading to recognition errors. This problem is exacerbated when recognizing compound characters, as the structural complexity is higher.

**Table 1:** Forming Bangla compound characters using two or more basic characters.

Combination of basic characters	Compound character
ব + দ	ব্দ
ম + প	ম্প
দ + ভ	দ্ভ
ক + ষ + ম	ক্ষ্ম

As a result, handwritten OCR for Bangla compound characters offers the possibility of achieving variability and accuracy for a deterministic pattern recognition problem. Therefore, this study focuses on the recognition of handwritten Bangla compound characters using a deep learning approach called CompoundDenseNet, which is proposed as a variant of DenseNet. DenseNet is a widely adopted model in the field of image recognition and classification, with applications ranging from OCR to medical imaging and beyond (Chatterjee et al., 2019; Alfaz et al., 2022c).

The BanglaLekha-Isolated (Biswas et al., 2017), Ekush (Rabby et al., 2018a), and CMATERdb (Sarkar et al., 2012) datasets were selected for this experiment due to their large collection of Bangla handwritten compound characters. These datasets capture a wide range of handwriting styles and real-world variations, ensuring better generalization for OCR models. Furthermore, they are widely used in the research community, making them ideal for evaluation and comparison in Bangla compound character recognition. Notably, BanglaLekha-Isolated stands out for its diverse set of handwritten characters, which are non-centered and non-uniform, unlike other datasets (Khan et al., 2022). Moreover, to assess the influence of different optimization strategies on model performance, the CompoundDenseNet architecture was evaluated using four well-established optimizers: SGD, Adam, RMSprop, and Adagrad. This analysis is essential, as each optimizer employs distinct approaches to gradient updates and learning rate adaptation, which can notably affect convergence behavior and overall accuracy. These optimizers were selected based on their widespread adoption and demonstrated effectiveness across a range of deep learning tasks, thereby providing a comprehensive understanding of the model's training dynamics. After conducting the experiment, the performance is evaluated using widely recognized metrics, including precision, recall, F1-score, and accuracy.

In summary, the primary objectives of this study are:

- To develop a new deep learning architecture, CompoundDenseNet, enhancing the accuracy of current state-of-the-art recognition techniques for Bangla handwritten compound character recognition.
- To test the performance of CompoundDenseNet using three widely used and diverse datasets, along with four well-known optimizers.
- To compare the performance of the CompoundDenseNet with existing supervised learning methods for Bangla handwritten compound character recognition.

The remainder of this paper is arranged as follows. Related Study introduces related works previously conducted on handwritten compound character recognition in Bangla. Proposed Methodology discusses the overall methodology of this study, including the data acquisition, data preprocessing, and details of the network architecture. The experimental results are discussed in detail in Experiment Details, Result Analysis and Discussion. Finally, Conclusion and Future Work concludes the paper by discussing conclusions and future work.

## 2 Related study

Numerous studies have investigated the recognition of handwritten Bangla numerals and basic characters, but the domain of handwritten compound character recognition remains relatively underexplored. The inherent complexity of Bangla compound characters, characterized by their structural similarities and morphological diversity, makes their recognition a challenging task (Bhattacharyya et al., 2022). Early efforts focused primarily on manual feature extraction, which is labor-intensive and time-consuming (Khan et al., 2022). Traditional machine learning methods often struggle due to the vast number of characters and their complex shapes. Recent advances in deep learning have introduced more efficient approaches to address these challenges.

Saha and Rahman (2024) present a novel approach called BanglaNet for Bangla handwritten character recognition using an ensemble of multiple CNNs, including Inception, ResNet, and DenseNet. This ensemble method aims to improve the accuracy of classifying Bangla basic characters, numerals, and modifiers by leveraging the strengths of different CNN architectures. The model is evaluated on three widely-used Bangla handwriting datasets: CMATERdb, BanglaLekha-Isolated, and Ekush, achieving baseline accuracies of 98.40%, 97.65%, and 97.32%, respectively. However, the authors do not provide specific accuracy measurements for compound character recognition, which is a key limitation given the complexity of these characters. Compound characters involve intricate patterns, and the paper lacks an assessment of how the model performs with them. Additionally, while high accuracy is reported on the datasets, the paper does not address the model's performance in real-world scenarios, particularly with noisy, distorted, or poorly written handwriting.

Haque et al. (2024) proposed an ensemble model with multi-channel attention for recognizing Bangla handwritten characters using the CMATERdb dataset, achieving 98% accuracy for both basic and compound characters. However, the use of multi-channel attention and ensemble models introduces additional computational complexity, which could be a limitation for real-time recognition or deployment on low-resource devices. While the model demonstrates high accuracy on the preprocessed dataset, it does not provide a separate evaluation specifically for compound characters. Additionally, the preprocessing steps, while beneficial for improving performance, may lead to increased computational time.

Tani et al. (2023) used an autoencoder to filter erroneous images from the Ekush dataset and then employed ResNet-50 for classification, achieving 97.92% accuracy. Similarly, Rakshit et al. (2023) evaluated multiple CNN models on the Ekush dataset, with ResNet152V2 and Xception achieving accuracies of 96.68% and 96.66%, respectively. Asraful et al. (2023) implemented a CNN-based approach on Ekush and BanglaLekha-Isolated datasets, achieving 92.48% and 97.24% accuracy, respectively.

Chakraborty et al. (2022) proposed a deep CNN evaluated on CMATERdb and BanglaLekha-Isolated datasets. Despite achieving a testing accuracy of 96.17% on CMATERdb after data augmentation, the model suffered from significant underfitting on the BanglaLekha-Isolated dataset, with testing accuracy dropping to 84.53%.

Islam M. S. et al. (2022) introduced RATNet, which achieved accuracies of 93.74% and 97.70% on BanglaLekha-Isolated and CMATERdb datasets, respectively. Fairiz Raisa et al. (2021) proposed a hybrid CNN-BiLSTM model, achieving 96.07% on CMATERdb but a lower 89.61% on BanglaLekha-Isolated. Mashrukh Zayed et al. (2021) employed a DCNN model, achieving accuracies of 93.26% on CMATERdb and 90.38% on BanglaLekha-Isolated. Kibria et al. (2020) utilized a Support Vector Machine (SVM) approach with features like Histogram of Oriented Gradients (HOG), achieving 89.73% accuracy on CMATERdb. Azad et al. (2020) proposed a Deep Convolutional Autoencoder Neural Network (DConvAENNet), which achieved 96.59% accuracy on the Ekush dataset, attributed to its larger sample size compared to CMATERdb and BanglaLekha-Isolated datasets. Abir et al. (2019) employed a CNN architecture with a fully connected network, achieving 89.30% accuracy on BanglaLekha-Isolated.

Several studies also explored segmentation-based approaches. Pramanik and Bag reduced classification complexity by segmenting compound characters, achieving 88.74% accuracy on CMATERdb (Pramanik and Bag, 2018). Sharif et al. integrated CNN with HOG features, achieving 92.50% accuracy on CMATERdb (Sharif et al., 2018). Roy et al. (2017) and Ashiquzzaman et al. (2017) developed CNN-based models achieving accuracies of 90.33% and 93.68% on CMATERdb, respectively, though both faced challenges with misclassification due to structural similarities.

Purkaystha et al. (2017) achieved 91.60% accuracy on BanglaLekha-Isolated using a DCNN model trained on 20 compound character classes. These findings underscore the effectiveness of deep learning in addressing the challenges of Bangla compound character recognition but also highlight the need for more robust models and diverse datasets to improve performance further.

Recent advancements in semi-supervised learning (SSL) have highlighted the importance of informative missingness, where the absence of labels can provide valuable insights into the data structure. In handwritten character recognition, missing strokes, partial characters, and ambiguous inputs are common, making informative missingness particularly relevant. Wu et al. (2025) discuss how informative missingness, where the missing data mechanism depends on observed features or class labels, can improve prediction performance by incorporating this missingness into the learning process. This approach, commonly modeled using MAR (Missing at Random) and MNAR (Missing Not at Random) mechanisms, can allow SSL algorithms to leverage unlabelled data more effectively, especially when the data is sparse or partially labeled. In the context of OCR, this framework can be used to address challenges related to missing or incomplete handwriting samples.

Despite notable advancements, existing approaches still face limitations such as overfitting, underfitting, and a lack of sufficient data diversity. These issues highlight the need for innovative model architectures and larger, more representative datasets. They also present significant opportunities for further research and development in Bangla handwritten compound character recognition. In addition, there is a strong need for a comprehensive study that deeply analyzes and discusses recognition performance specifically on Bangla handwritten compound characters. While this study focuses on advancing the CompoundDenseNet architecture and improving performance through innovative architectural choices and optimized training strategies, future research could explore the integration of informative missingness in semi-supervised learning. This approach could help the model better handle challenges such as missing strokes, partial characters, and ambiguous inputs, ultimately improving its robustness and generalization for real-world OCR applications.

The recent works on handwritten Bangla compound character recognition are summarized in Table 2. Most studies employ deep learning approaches, which outperform traditional feature extraction-based machine learning methods. Commonly used datasets include CMATERdb, BanglaLekha-Isolated, and Ekush. However, few studies focus specifically on compound character classification. Given the intricate shapes of compound characters, comprehensive evaluations focusing on these characters remain limited. Moreover, many studies do not assess their proposed methods across all three major datasets. Among the reviewed works, RATNet achieves the highest accuracy of 93.74% on the BanglaLekha-Isolated dataset, highlighting room for improvement in this area. DConvAENNet performs better with the Ekush dataset but exhibits lower accuracy on BanglaLekha-Isolated, likely due to non-centered and non-uniform images Khan et al., (2022). Additionally, there is a lack of research comparing optimizers or justifying their selection for compound character recognition. Table 3 outlines the advantages and limitations of these methods. This study addresses these gaps by developing a deep learning architecture to achieve higher accuracy for Bangla compound character recognition. It evaluates the model's performance across BanglaLekha-Isolated, Ekush, and CMATERdb datasets while analyzing the efficacy and rationale for various optimizers.

**Table 2:** An overview of some recent works on Bangla handwritten compound character recognition.

Study	Method	Dataset	Accuracy
Saha and Rahman (2024)	BanglaNet	BanglaLekha–Isolated	97.65%
		Ekush	97.32%
		CMATERdb	98.40%
Tani et al. (2023)	ResNet–50	Ekush	97.92%
Rakshit et al. (2023)	ResNet152V2	Ekush	95.64%
	Xception		95.58%
Asraful et al. (2023)	CNN	BanglaLekha–Isolated	92.48%
		Ekush	97.24%
Chakraborty et al. (2022)	DCNN	CMATERdb	96.17%
		BanglaLekha–Isolated	93.43%
Islam M. S. et al. (2022)	RATNet	CMATERdb	97.70%
		BanglaLekha–Isolated	93.74%
Fairiz Raisa et al. (2021)	CNN–BiLSTM	CMATERdb	96.07%
		BanglaLekha–Isolated	89.61%
Mashrukh Zayed et al. (2021)	DCNN	CMATERdb	93.26%
		BanglaLekha–Isolated	90.38%
Kibria et al. (2020)	Feature extraction and SVM	CMATERdb	89.73%
Azad et al. (2020)	DConvAENNet	CMATERdb	90.15%
		BanglaLekha–Isolated	93.36%
		Ekush	96.59%
Abir et al. (2019)	Multi–layer CNN	BanglaLekha–Isolated	89.30%
Pramanik and Bag (2018)	Chain code histogram feature set and MLP	CMATERdb	88.74%
Sharif et al. (2018)	HOG–CNN Hybrid Model	CMATERdb	92.50%
Roy et al. (2017)	DCNN	CMATERdb	90.33%
Ashiquzzaman et al. (2017)	DCNN with greedy layer–wise training	CMATERdb	93.68%
(Purkaystha et al. 2017)	DCNN	BanglaLekha–Isolated	91.60%

**Table 3:** Comparison of the proposed method with previous methods in terms of advantages and limitations.

Method	Advantage	Limitation
BanglaNet (Saha and Rahman, 2024)	Combining multiple CNN architectures allows the model to capture diverse and complementary features.	Ensembling boosts performance but increases computational cost, making it less practical for resource-limited settings.
ResNet–50 (Tani et al., 2023)	ResNets accelerate training with skip connections for efficient gradient propagation.	Hop connections in ResNets raise complexity, increasing computational and memory demands.
DCNN (Chakraborty et al., 2022; Mashrukh Zayed et al., 2021; Roy et al., 2017; Purkaystha et al., 2017)	Deep CNN boasts a straightforward architecture with easily adjustable layers and hyperparameter tuning process.	DCNNs demand a substantial amount of time and data for training and are prone to overfitting and vanishing gradient problems.
RATNet (Islam M. S. et al., 2022)	RATNet uses parameter-efficient dense blocks and reduces computational costs with a spatial attention module.	The spatial attention module in RATNet can increase complexity, introduce biases, and complicate training and optimization.
CNN–BiLSTM (Fairiz Raisa et al., 2021)	The vanishing gradient problem that might arise from using CNN is mitigated by BiLSTM.	The process of training the combination might be time-consuming and data intensive.
Feature extraction and SVM (Kibria et al., 2020)	SVMs have a lesser susceptibility to overfitting than neural networks.	SVMs are sensitive to noisy data and not suitable for multi-class problems.
DConvAENNet (Azad et al., 2020)	Autoencoders reduce dimensionality by capturing essential features and removing noise.	The training time required for an Autoencoder is longer compared to that of a DCNN model for training images.
Multi–layer CNN (Abir et al., 2019)	The inception module used with CNN enhances the overall efficiency of the model by extracting hierarchical features.	Simple CNNs are slow; adding inception modules boosts structural complexity.
Chain code histogram feature set and MLP (Pramanik and Bag, 2018)	The MLP incorporates additional hidden layers and functions for activation, therefore providing non-linearity and enhancing its adaptability.	Convergence necessitates the inclusion of additional parameters, image data, and a longer duration in MLP.
HOG–CNN Hybrid Model (Sharif et al., 2018)	Using HOG and CNN reduces training time compared to traditional networks.	Complex integration, increased computational cost, resource-intensive training, intricate fine-tuning, and requires large dataset.
ResNet152V2 (Rakshit et al., 2023)	ResNets enhance training efficiency with gradient-propagating skip connections.	Hop connections in ResNets raise complexity, increasing computational and memory demands.
Xception (Rakshit et al., 2023)	Xception uses depthwise separable convolutions for superior parameter efficiency.	Complex architecture demands time and resources for training.

## 3 Proposed methodology

**Figure 1:**
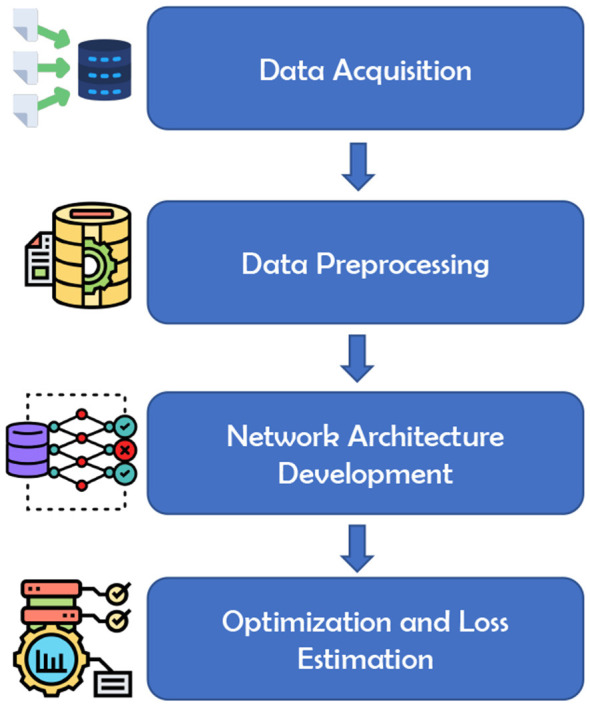
Overview of the proposed methodology for Bangla handwritten compound character recognition.

This section thoroughly explains the proposed methodology for recognizing handwritten Bangla compound characters. The methodology starts with the data acquisition phase from the Banglalekha-Isolated dataset. The data acquisition phase is followed by a data preprocessing phase. After data pre-processing, a new deep learning architecture based on DenseNet is developed to extract features from the dataset to recognize handwritten Bangla compound characters with higher accuracy. Overall, the recognition method consists of four phases, namely (*i*) data acquisition, (*ii*) data preprocessing, (*iii*) network architecture development and (*iv*) optimization and loss estimation. An overview of the proposed methodology can be found in Figure 1. These phases are discussed in detail in the following sections.

### 3.1 Data acquisition

For the purpose of consistent comparison with existing models, this study evaluates the performance of the proposed CompoundDenseNet architecture using three widely-used datasets in the research community: BanglaLekha-Isolated, Ekush, and CMATERdb. The BanglaLekha-Isolated dataset contains 24 compound characters, which were chosen to maintain consistency with prior research. From the Ekush and CMATERdb datasets, we selected 50 compound characters that were common across both datasets to enable a fair and meaningful comparison. These selections were made based on the need for comparability and representativeness of the compound characters across different handwriting styles and datasets.

**Figure 2:**
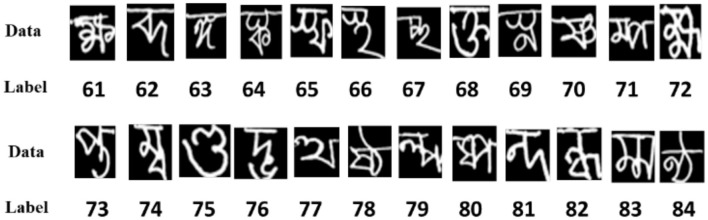
Compound characters with corresponding labels.

The BanglaLekha-Isolated dataset (Biswas et al., 2017) contains 50 handwritten Bangla basic characters, 10 numerals, and 24 selected compound characters, totaling 166,105 samples. Each compound character includes approximately 2,000 handwritten instances collected from male and female participants aged 6 to 28, representing various regions of Bangladesh. As noted by the authors, this dataset is among the largest collections of handwritten Bangla characters and is particularly valuable for studying handwriting variations across age and gender. However, the sample distribution is not uniform across all characters. For this study, only the 24 handwritten Bangla compound characters—labeled with identifiers 61–84, as shown in Figure 2—were extracted to construct a focused dataset tailored to the research objectives.

The Ekush (Rabby et al., 2018a) dataset is a large, multipurpose Bangla handwritten character database containing 367,018 isolated character instances across 122 distinct character classes, including 50 compound characters. These compound characters represent a wide variety of handwriting styles from real individuals, making the dataset highly valuable for evaluating models on diverse writing patterns.

Similarly, the CMATERdb dataset (Sarkar et al., 2012) is a widely recognized benchmark in Bangla OCR research. While CMATERdb includes over 100 compound character classes, we selected 50 compound characters that are common with those in the Ekush dataset to ensure a fair and consistent comparison. The CMATERdb dataset is particularly valuable due to its structured format and public availability, making it an important resource for evaluating OCR systems. Its inclusion in this study helps evaluate the model across a broad range of handwriting styles and character types, providing a comprehensive performance benchmark.

### 3.2 Data pre-processing

**Figure 3:**
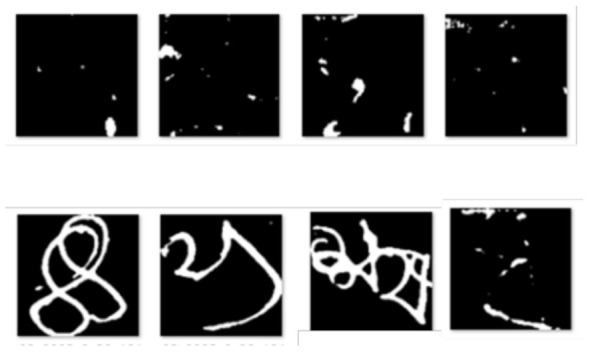
Erroneous data in BanglaLekha–Isolated repository.

Several blank and erroneous images were identified in the BanglaLekha-Isolated repository during the data acquisition phase, as illustrated in Figure 3. To address this, the dataset was manually cleaned by removing all blank and misplaced images from each class. After cleaning, the dataset was divided into two parts: one for training and the other as an unseen validation set. The number of samples in each class and their corresponding labels are shown in Table 4.

**Table 4:** Data distribution.

Label	Compound character	No. of train samples	No. of validation samples
61	ক্ষ	590	148
62	ব্দ	599	150
63	ঙ্গ	539	135
64	স্ক	543	136
65	স্ফ	517	129
66	স্থ	594	149
67	চ্ছ	539	135
68	ক্ত	594	149
69	স্ন	506	124
70	ষ্ণ	534	133
71	ম্প	534	133
72	হ্ম	506	127
73	প্ত	514	151
74	ম্ব	622	129
75	ণ্ড	564	141
76	দ্ভ	466	116
77	ন্থ	560	140
78	ষ্ঠ	555	139
79	ল্প	558	140
80	ষ্প	554	138
81	ন্দ	607	152
82	ন্ধ	586	115
83	ম্ম	622	148
84	ন্ঠ	491	123

Since one of the key potentials of the proposed method is its use in model training for script assessment, the presence of blank or erroneous images could lead to increased misclassification rates. Therefore, their removal was essential. Additionally, the original sample sizes in the BanglaLekha-Isolated dataset were inconsistent. To ensure uniformity and improve efficiency, all images were resized to 32 × 32 pixels and converted into three-channel format, significantly reducing computational time and memory usage. Finally, pixel values were normalized to fall within the range [0, 1]. This preprocessing ensured uniformity and reduced misclassification caused by erroneous data.

**Figure 4:**
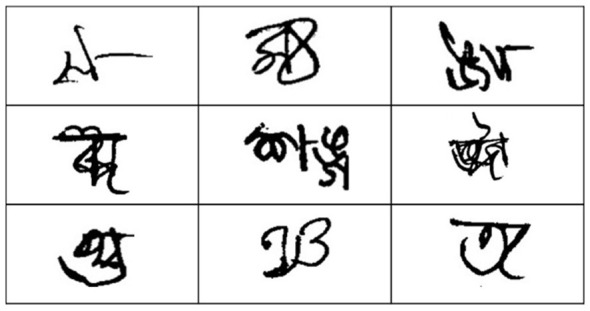
Erroneous data in Ekush repository.

**Figure 5:**
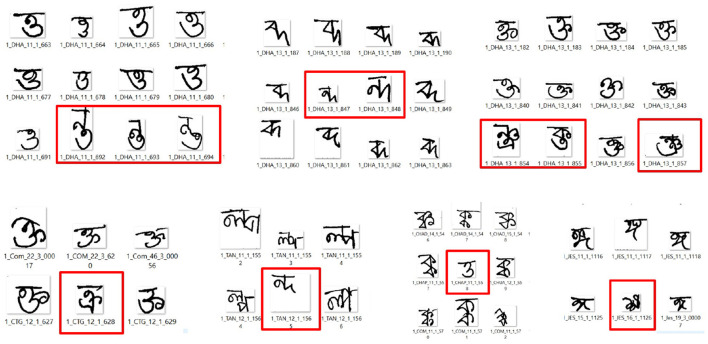
Misallocated data in Ekush repository.

In the Ekush dataset, no blank images and only a negligible number of illegible or poorly written samples were found. However, a notable number of misallocated samples were present, as shown in Figures 4, 5. These outliers were removed to improve the reliability of both training and prediction. The remaining samples were resized to 32 × 32 pixels and normalized following the same procedure used for the BanglaLekha-Isolated dataset.

The CMATERdb dataset, although free from illegible or misallocated samples, posed a challenge due to its limited number of samples per class (100–150). To address this, 50 compound characters overlapping with those in the Ekush dataset were selected, and data augmentation techniques were applied to address class imbalance. The augmented samples were then resized to 32 × 32 pixels and normalized before training.

### 3.3 CompoundDenseNet development

The proposed network architecture is inspired by Huang's DenseNet (Huang et al., 2017), which is well-known for its densely connected structure, where each layer receives input from all preceding layers in a feed-forward manner. DenseNet's architecture, with *L* layers, establishes 1/2*L*(*L*+1) direct connections, significantly reducing the distance between the input and output layers. This structure enhances training efficiency and accuracy by mitigating the vanishing gradient problem and promoting feature reuse (Huang et al., 2017). Compared to conventional CNNs, DenseNet achieves faster convergence and higher classification accuracy while using fewer parameters (Alom et al., 2018). Its architecture comprises multiple Dense Blocks interconnected by Transition Layers, where each Transition Layer facilitates efficient flow of information between adjacent Dense Blocks.

In this network, the *L*^th^ layer accepts all concatenated features *f*_0_, *f*_1_, *f*_2_......, *f*_L − 1_ from the foregoing layers as input, which is represented in the following expression with a single tensor *HL*(.).


$$\begin{array}{l} f\text{\_}L=H\text{\_}L\left(\left[f\text{\_}0,f\text{\_}1,f\text{\_}2,f\text{\_}3,......,f\text{\_}L-1\right]\right) \end{array}$$ (1)

The proposed CompoundDenseNet is particularly derived from the DenseNet-121 architecture (Huang et al., 2017), selected as the baseline because of its well-established effectiveness in feature reuse and gradient propagation. Instead of using the architecture without modification, we iteratively adjusted it based on empirical testing to adapt it to the complexities of Bangla handwritten compound character recognition. Specifically, key parameters such as the growth rate, number of dense blocks, and block repetitions were adjusted while monitoring the model's accuracy and overfitting behavior.

**Figure 6:**
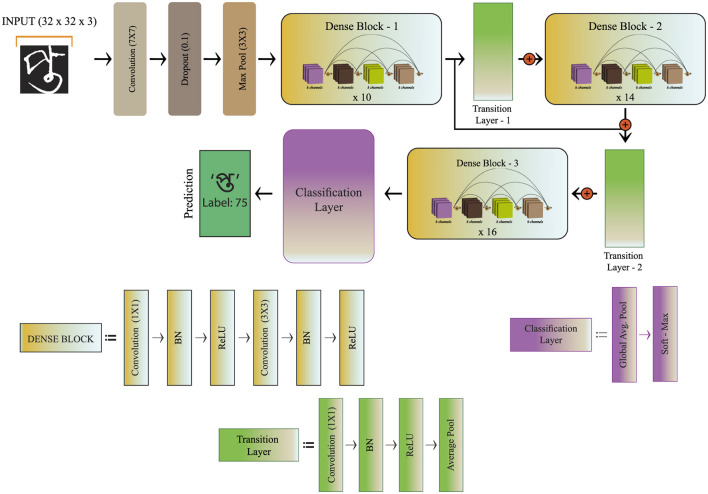
CompoundDenseNet architecture.

The final configuration, consisting of three dense blocks with repetitions of 10, 14, and 16, and a growth rate, *k* of 12, was chosen because it provided the best balance between classification accuracy and computational efficiency, as shown in Figure 6 and the corresponding performance graphs. The layers in a single Dense Block are as follows: a convolution layer with a kernel size of 1 × 1 and a filter count of four times the growth rate, a batch normalization (BN) layer, an activation layer, a convolution layer with a kernel size of 3 × 3 and a filter count equal to the growth rate, a further BN layer, and another activation layer. In the proposed study, Rectified Linear Unit (ReLU) has been employed in the activation layer. While ReLU has been around since (Hahnloser et al., 2000), its prominence as an activation function in neural network architecture has been increasing due to its numerous advantages (Albawi et al., 2017). Compared to other commonly used activation functions such as sigmoid and *tanh*, where both might result in disappearing gradient issues in deeper network architecture, ReLU features a gradient that is constant for the positive input, and the zero-gradient produced by the ReLU leads to a sparser representation that overcomes disappearing gradient problem and provides improved training. The equation of ReLU is expressed in Equation 1, which produces 0 when a is less than 0 and a linear output when a is greater than or equal to zero.


$$\begin{array}{l} f\left(a\right)=\max\left(0,a\right) \end{array}$$ (2)

The purpose of using pooling expressed in Equation 2 is to gradually lower the dimensionality of the convolved output in order to minimize the number of parameters and optimize computation. It reconfigures the feature representation to extract important features from the combined feature representation and discard unnecessary features (Alfaz et al., 2022b). Regarding this, max pooling and average pooling have been integrated into the design. The input is initially passed to a convolution layer having kernel size 7 × 7, stride 2, and filters two times the growth rate, followed by a 10 % dropout layer and a max pooling layer. The three Dense blocks, namely Dense Block-1, Dense Block-2, and Dense Block-3, repeat 10, 14, and 16 times, respectively. There are two transition layers in the proposed architecture, namely Transition Layer-1 and Transition Layer-2. Each Transition Layer comprises a single convolution layer where the kernel size is 1 × 1, and the number of filters is half of the total filters of its prior Dense Block, followed by the activation function ReLU and an average pooling layer. The compression factor θ used in the transition layers to reduce the feature maps of the proposed network is 0.5. The Global Average Pooling is located after the Dense Block-3, and the soft-max function is attached to the last layer for classification.

**Figure 7:**
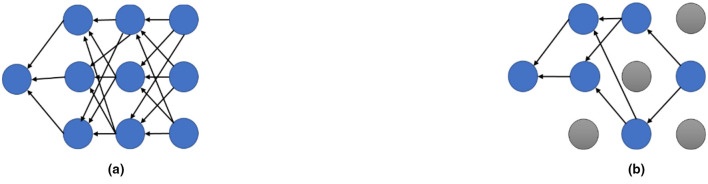
Effect of dropout in a standard neural network. **(a)** A standard neural network. **(b)** After application of dropout.

To enhance generalization and prevent overfitting or underfitting, the proposed network incorporates two regularization techniques: BN and Dropout. These methods are widely recognized for addressing long training times in multi-layer neural networks (Garbin et al., 2020). Dropout combats overfitting by randomly deactivating neurons and their connections during training, as illustrated in Figure 7. This process reduces reliance on specific neurons, promotes robustness, and encourages the network to learn more generalized features (Srivastava et al., 2014). By repeatedly disabling units, Dropout lowers the error rate and improves overall network performance. The dropout rate was selected based on initial experiments that demonstrated a clear divergence between training and validation accuracy when no dropout was applied. As discussed in the following analysis, this overfitting issue necessitated the integration of a dropout layer to stabilize the network and improve overall generalization performance.

The variation in input values across network layers can increase training time. BN addresses this issue by normalizing the inputs of each layer, thereby reducing training time (Garbin et al., 2020). BN also introduces noise during training, which aids in regularizing the network. When combined with Dropout, BN not only accelerates convergence but also enhances the overall training efficiency. By mitigating the internal covariate shift, BN allows for a more stable learning process, reducing the need for higher Dropout rates. The summary of the proposed CompoundDenseNet architecture is presented in Table 5.

**Table 5:** Summary of the CompoundDenseNet architecture.

Layers	Output size	Proposed CompoundDenseNet
Convolution	16 × 16	7 × 7 kernel, stride 2
Dropout	16 × 16	10%
Max pooling	8 × 8	3 × 3 kernel, stride 2
Dense block–1	8 × 8	Conv1×1Conv3×3×10
Transition layer–1	8 × 8	Conv 1 × 1
	4 × 4	2 × 2 average pooling, stride 2
Dense Block–2	4 × 4	Conv1×1Conv3×3×14
Transition layer–2	4 × 4	Conv 1 × 1
	2 × 2	2 × 2 average pooling, stride 2
Dense block–3	2 × 2	Conv1×1Conv3×3×16
Classification layer	1 × 1	Global average pool
		Dense fully–connected, softmax
Total parameters		≈0.57M
Total training time		≈28 mins

### 3.4 Optimization and loss estimation

This research examines the effectiveness of four commonly utilized optimization algorithms—SGD, AdaGrad, RMSProp, and Adam (Haji and Abdulazeez, 2021)—on the BanglaLekha-Isolated dataset, employing their default learning rates. The optimizer achieving the best results in terms of training efficiency, convergence stability, and classification accuracy was selected for additional testing on two other datasets.

SGD is well-suited for optimizing large-scale neural networks by processing randomly chosen subsets of data during each iteration (Haji and Abdulazeez, 2021). By updating parameters incrementally, it enhances memory utilization and facilitates faster convergence compared to alternative algorithms.

AdaGrad modifies the learning rate individually for each parameter, decreasing it for parameters updated frequently (Haji and Abdulazeez, 2021). This property is particularly advantageous for datasets with both sparse and dense features, enabling more effective learning.

RMSProp, a widely adopted adaptive stochastic approach, excels at minimizing loss by maintaining an exponentially weighted moving average of previous squared gradients, which aids in efficient convergence to local minima (Alfaz et al., 2022b).

Adam, a sophisticated extension of SGD, dynamically adjusts learning rates and weights to optimize the network (Kingma and Ba, 2014). Known for its computational simplicity and rapid convergence, Adam is particularly effective during early training stages. It also adapts learning rates across layers, improving overall performance.

Categorical Cross-Entropy (Rabby et al., 2018b) was used as the loss function for this multi-class classification task. Its convex and smooth properties simplify gradient-based optimization, ensuring efficient minimization and robust convergence.

## 4 Experiment details, result analysis and discussion

An experiment is conducted, followed by an evaluation to evaluate the proposed methodology. Experiment Details discusses the experimental setup and evaluation metrics. Experimental Result Analysis and Discussion presents the analysis of the experimental results. The evaluation metrics are used to evaluate the results, and a comparison is also made with the relevant existing approaches.

### 4.1 Experiment details

The experimental setup and the evaluation metrics used to evaluate the efficacy of the developed deep learning architecture are discussed in detail in this section. Sections Experimental Setup and Evaluation Metrics provide the experimental setup and evaluation metrics, respectively.

#### 4.1.1 Experimental setup

All the experiments have been carried out on an x64-based system, which is the Alienware m15 R6, equipped with an 11th Generation Intel(R) Core(TM) i7-11800H processor and an NVIDIA GeForce RTX 3060 graphics processing unit.

#### 4.1.2 Evaluation metrics

To further evaluate the performance of the proposed architecture, some evaluation metrics are used. In the assessment phase, in addition to the training result and validation result, the confusion matrix, accuracy, precision, recall, and F1-Score are utilized to evaluate the recognition performance of the proposed deep learning architecture. These evaluation metrics are well-known and widely used in various fields for evaluating various machine learning algorithms (Islam S. S. et al., 2022; Alfaz et al., 2022a; Hasan et al., 2023) for prediction or classification problems, information extraction, and recommender systems (Sarwar et al., 2021; Sarwar and Noor, 2021) algorithms and many more.

**Figure 8:**
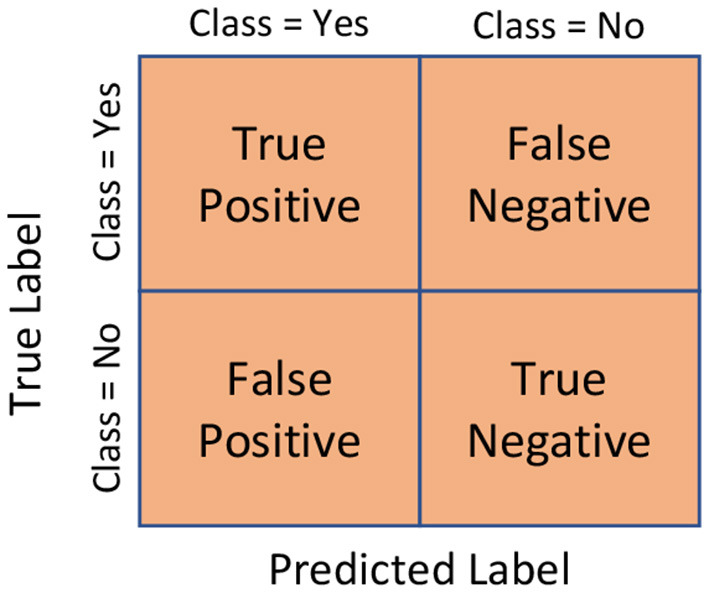
An example of a confusion matrix for a two-class classifier.

The confusion matrix is used to display and summarize the performance of the suggested classifier by comparing the actual labels to the predicted labels. Moreover, it provides insight into the types of errors generated by the classification model. An example of a confusion matrix for a two-class classifier is presented in Figure 8. True positives (*TP*_*o*_) and true negatives (*TN*_*e*_) denote the correctly classified labels. On the other hand, false positives (*FP*_*o*_) and false negatives (*FN*_*e*_) denote incorrectly classified labels, indicating that the predicted class contradicts the actual class.

The most intuitive performance evaluation metric for the developed deep learning architecture is to measure its accuracy. Accuracy is simply the proportion of correctly classified compound characters to the total number of compound characters. The higher the accuracy, the more effective the architecture is. The accuracy can be calculated by Equation 3.


$$\begin{array}{l} Accuracy=\frac{\left(TP_{o}+TN_{e}\right)}{\left(TP_{o}+TN_{e}+FP_{o}+FN_{e}\right)} \end{array}$$ (3)

Here, (*TP*_*o*_+*TN*_*e*_) denotes the correctly classified compound characters and (*TP*_*o*_+*TN*_*e*_+*FP*_*o*_+*FN*_*e*_) denotes the total number of compound characters.

Precision refers to the proportion of correctly classified compound characters among all instances predicted as compound characters. A well-performing classifier should yield a precision value close to 1. The precision is calculated using Equation 4.


$$\begin{array}{l} Precision=\frac{TP_{o}}{\left(TP_{o}+FP_{o}\right)} \end{array}$$ (4)

Here, *TP*_*o*_ denotes the correctly classified compound characters, and *FP*_*o*_ denotes the incorrectly classified compound characters as correct.

Recall is the proportion of correctly classified compound characters to the total number of actual compound characters in the ground truth. If the classifier performs well, the recall value should be close to or equal to 1. Recall has been calculated using Equation 5.


$$\begin{array}{l} Recall=\frac{TP_{o}}{\left(TP_{o}+FN_{e}\right)} \end{array}$$ (5)

Here, *TP*_*o*_ denotes the correctly classified compound characters, and *FN*_*e*_ denotes the compound characters which are actually correct but classified as incorrect.

The weighted mean of Precision and Recall is the F1-Score. Therefore, both false positives and false negatives are included while calculating this score. F1-Score is often more beneficial than accuracy, particularly if there is an unbalanced distribution of classes. The F1-Score can be calculated using Equation 6.


$$\begin{array}{l} F1-Score=2\times \frac{Precision*Recall}{Precision+Recall} \end{array}$$ (6)

### 4.2 Experimental result analysis and discussion

The BanglaLekha-Isolated dataset was used to evaluate the performance of the optimizers discussed in Section 3.4. The dataset was split into 13,294 samples for training and 3,280 samples for validation, with each sample resized to 32 × 32 × 3. Table 6 illustrates variations in writing styles for Bangla compound characters in the validation set. The model was trained with a batch size of 20 for 50 epochs.

**Table 6:** Few instances of validation data in various forms of writing.

Compound character	Sample 1	Sample 2	Sample 3	Sample 4
ক্ষ	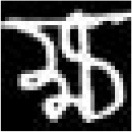	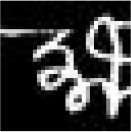	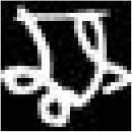	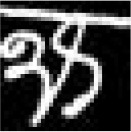
ঙ্গ	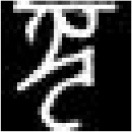	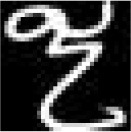	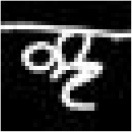	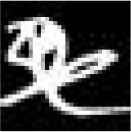
স্থ	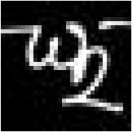	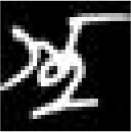	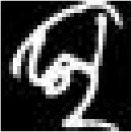	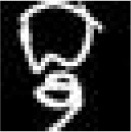
ষ্ণ	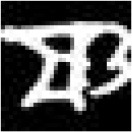	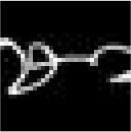	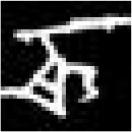	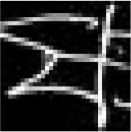
ম্প	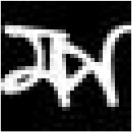	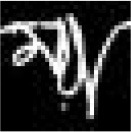	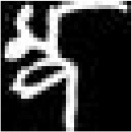	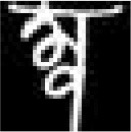
হ্ম	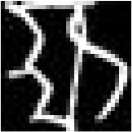	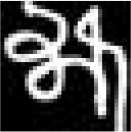	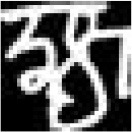	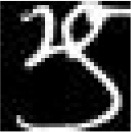

**Figure 9:**
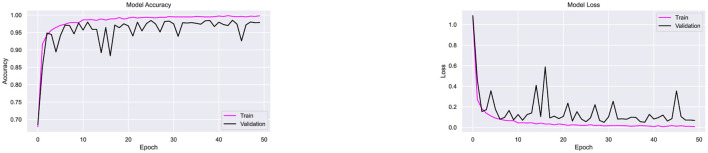
Performance testing of CompoundDenseNet without the inclusion of Dropout, showing both the training and validation accuracy and loss curves.

Dropout regularization was incorporated iteratively during experimentation. Initially, the architecture, which includes batch normalization, was tested using the Adam optimizer without dropout. As shown in Figure 9, the model achieved 97.9% accuracy but exhibited fluctuations in accuracy and loss, indicating overfitting. To address this, a dropout rate of 0.1 was introduced, and subsequent optimizer testing experiments were conducted. To ensure consistency and fair comparison across all experiments, the training conditions were kept the same. The batch size and the number of epochs were fixed at 20 and 50, respectively, for all optimizers and datasets. The image size was set to 32 × 32 pixels for all experiments. This approach allowed any performance differences between the optimizers to be attributed solely to the optimizer's properties, rather than to variations in the training setup.

**Figure 10:**
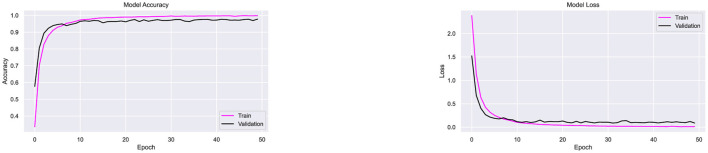
Performance testing of CompoundDenseNet using the SGD optimizer, showing both the training and validation accuracy and loss curves.

The performance evaluation began with the SGD optimizer, which demonstrated excellent classification accuracy of 97.7% on the CompoundDenseNet architecture. Figure 10 shows the training and validation accuracy and loss curves. The model correctly classified 3,205 out of 3,280 Bangla compound character images in the validation set, with a final validation loss of 0.088. The decreasing loss curve in Figure 10, reflects effective optimization and strong generalization, with minimal signs of overfitting. The stable loss at the end of training further indicates robust convergence.

**Figure 11:**
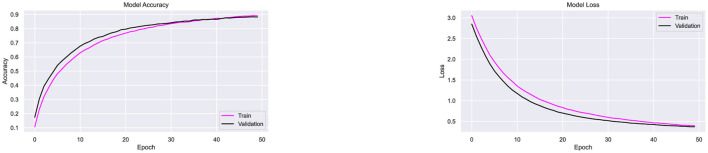
Performance testing of CompoundDenseNet using the AdaGrad optimizer, showing both the training and validation accuracy and loss curves.

After training CompoundDenseNet with the SGD optimizer, the AdaGrad optimizer was evaluated. Figure 11 shows the accuracy and loss curves during training and validation. AdaGrad achieved a moderate classification accuracy of 88.20%, with a high validation loss of 0.37. Out of 3,280 Bangla compound character images, the model correctly classified 2,893. Although the accuracy curve in Figure 11 shows gradual improvement, the optimizer exhibited slower convergence, resulting in extended training time to achieve reasonable accuracy.

**Figure 12:**
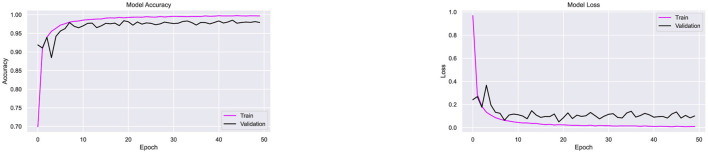
Performance testing of CompoundDenseNet using the RMSProp optimizer, showing both the training and validation accuracy and loss curves.

The model was further trained using the RMSProp optimizer, yielding excellent results. The model achieved a classification accuracy of 97.9% with a validation loss of 0.085, correctly classifying 3,212 out of 3,280 Bangla compound character images. Figure 12 illustrates the accuracy and loss curves during training and validation. The curves exhibit minimal fluctuations, indicating strong generalization and a low likelihood of overfitting. Additionally, the stable loss and high accuracy on both training and validation data highlight effective convergence.

The optimizer evaluation concluded with the model using the Adam optimizer, achieving exceptional results. The model attained 99.59% training accuracy, 98.50% validation accuracy, 0.013 training loss, and 0.049 validation loss. As the best-performing optimizer, Adam was adopted for subsequent experiments. Despite challenges such as complex writing styles and structural similarities among certain characters, the model demonstrated robust classification, achieving 98.50% accuracy. Although CompoundDenseNet is deep and complex, the average training time of 28 min demonstrates that the model has been optimized to strike a balance between performance and computational efficiency. Additionally, the model's relatively low parameter count of approximately 0.57 million ensures lower memory consumption and reduced computational resource requirements, which helps reduce training time compared to more complex models. This compact design makes it more feasible for deployment in real-world OCR applications, particularly on devices with limited resources, such as mobile phones or embedded systems.

**Figure 13:**
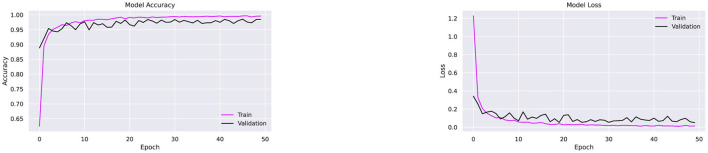
Performance testing of CompoundDenseNet using the Adam optimizer, showing both the training and validation accuracy and loss curves.

Figure 13 illustrates strong generalization with minimal overfitting or underfitting. Faster convergence is evident from the rapid decline in loss, as shown in Figure 13. The incorporation of dropout effectively reduced overfitting and improved accuracy compared to the setup without regularization.

**Figure 14:**
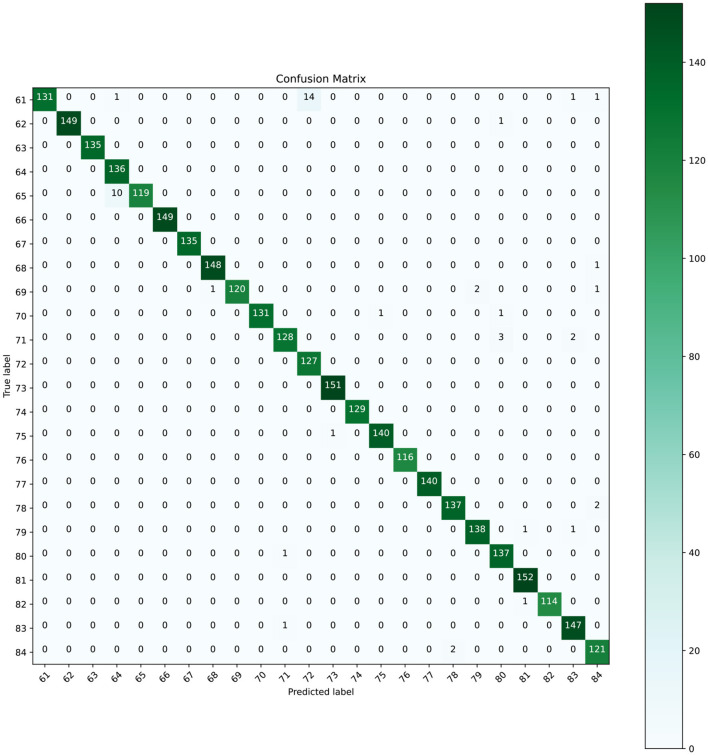
Confusion matrix for the BanglaLekha–Isolated dataset.

**Figure 15:**
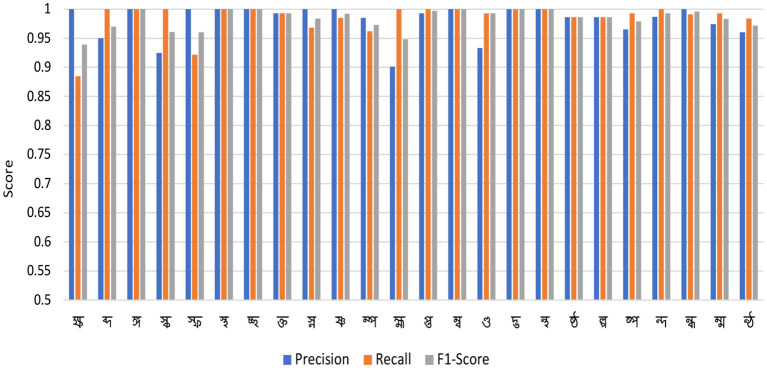
Precision, recall, and F1-score for 24 classes in the BanglaLekha–Isolated dataset.

According to Table 5, the proposed model utilizes a minimal number of parameters and requires relatively short training time. However, misclassifications were observed for structurally similar characters such as “ক্ষ” and “হ্ম”, or “প্ত” and “ণ্ড”, which are challenging even for humans to distinguish in handwritten form. As shown in Figure 14, the confusion matrix indicates that the model correctly classified all samples of characters such as “ঙ্গ”, “স্থ”, “চ্ছ”, “ম্ব”, “দ্ভ”, and “ন্থ”. The classification metrics, including precision, recall, and F1-score, are summarized in Table 7, where these characters achieved perfect scores. A graphical representation of the classification results across all 24 classes is illustrated in Figure 15.

**Table 7:** Performance evaluation of class-wise classification on the BanglaLekha–Isolated dataset.

Label	Compound character	Precision	Recall	F1-score	Support
61	ক্ষ	1.000	0.885	0.939	148
62	ব্দ	1.000	0.993	0.997	150
63	ঙ্গ	1.000	1.000	1.000	135
64	স্ক	0.925	1.000	0.961	136
65	স্ফ	1.000	0.922	0.960	129
66	স্থ	1.000	1.000	1.000	149
67	চ্ছ	1.000	1.000	1.000	135
68	ক্ত	0.993	0.993	0.993	149
69	স্ন	1.000	0.968	0.984	124
70	ষ্ণ	1.000	0.985	0.992	133
71	ম্প	0.985	0.962	0.973	133
72	হ্ম	0.901	1.000	0.948	127
73	প্ত	0.993	1.000	0.997	151
74	ম্ব	1.000	1.000	1.000	129
75	ণ্ড	0.993	0.993	0.993	141
76	দ্ভ	1.000	1.000	1.000	116
77	ন্থ	1.000	1.000	1.000	140
78	ষ্ঠ	0.986	0.986	0.986	139
79	ল্প	0.986	0.986	0.986	140
80	ষ্প	0.965	0.993	0.979	138
81	ন্দ	0.987	1.000	0.993	152
82	ন্ধ	1.000	0.991	0.996	115
83	ম্ম	0.974	0.993	0.983	148
84	ন্ঠ	0.960	0.984	0.972	123
Accuracy				0.985	3280
Weighted average		0.985	0.985	0.985	3280

However, the misclassified instances from the BanglaLekha-Isolated dataset are also visible in the confusion matrix and presented in Table 8. The proposed CompoundDenseNet demonstrates the lowest classification accuracy for “ক্ষ”, as 17 out of 148 unseen samples are erroneously labeled while 14 are classified as “হ্ম”. Besides, the architecture shows the second lowest classification accuracy for label 65 “স্ফ” where 10 out of 129 instances are wrongly classified as label 64 “স্ক”. Table 8 demonstrates that some of the validation samples present in label 61 look more like label 72 and vice versa. Similarly, the unseen samples of label 80 look like label 71. Thus, the reason for the misclassification rate largely depends on the writing style, as people also sometimes get confused in recognizing handwritten Bangla characters due to the complex cursive writing style. Table 8 shows some instances in the validation data that look more identical to the character that the architecture recognizes. Therefore, a more well-founded dataset can provide higher classification accuracy. Besides, overwriting and unintentional strokes also contributed to noise that led to misidentification.

**Table 8:** Some misclassified instances.

Test data	True character	True label	Identified character	Identified label
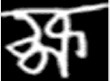	ক্ষ	61	হ্ম	72
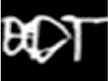	ম্প	71	ষ্প	80
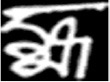	হ্ম	72	ক্ষ	61
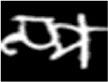	ম্প	71	ল্প	79
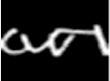	ষ্প	80	ম্প	71
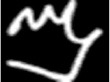	ণ্ড	75	ক্ত	68
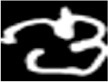	ণ্ড	75	প্ত	73

An analysis of the confusion matrix revealed that characters such as “ক্ষ” and “স্ফ” are frequently misclassified due to their structural similarity. This issue arises from intrinsic difficulties in distinguishing these characters, which can also be challenging for human annotators. These misclassifications are also influenced by the variability in handwriting styles within the dataset. Addressing these challenges will require improving preprocessing steps or employing more advanced architectures that can capture subtle visual differences between such similar compound characters.

Table 9 presents the performance of some previously used deep learning architectures along with the proposed architecture for the BanglaLekha-Isolated dataset. From Table 9, it is visible that the proposed DenseNet architecture in this study outperforms all the recent works done on the Banglalekha-Isolated dataset to recognize Bangla handwritten compound characters. The proposed architecture shows better accuracy than some of the well-known deep learning architecture, namely VGG-16, ResNet-50, DCNN, DenseNet-121, and LeNet-5 mentioned in Table 9.

**Table 9:** Performance comparison of some existing studies on compound character recognition using the BanglaLekha–Isolated dataset.

Study references	Method	Accuracy (%)
Saha and Rahman (2024)	BanglaNet	97.65
Islam M. S. et al. (2022)	LeNet–5	90.58
	VGG–16	93.18
	ResNet–50	93.41
	RATNet–No–SAM–RB	92.24
	RATNet	93.74
	DenseNet–121	93.52
Asraful et al. (2023)	CNN	92.48
Fairiz Raisa et al. (2021)	CNN–BiLSTM	89.61
Mashrukh Zayed et al. (2021)	DCNN	90.38
Azad et al. (2020)	DConvAENNet	93.36
Abir et al. (2019)	CNN with inception	89.30
Purkaystha et al. (2017)	DCNN	91.60
Rabbi et al. (2022)	KDANet	98.07
Chakraborty et al. (2022)	DCNN with augmentation	92.89
Jaman et al. (2022)	CNN	77.55
This study	Proposed CompoundDenseNet	98.50

The optimizer plays an important role in the overall performance of the model by ensuring convergence, adaptability, robustness and stability in the network. The research studies mentioned in Table 9 have not investigated the effect of different optimizer in the network before combining it with their proposed network. However, this proposed study has illustrated a thorough analysis on the performance of different optimizer then one optimizer has been selected. Besides, this study analyse the performance of a widely used regulizer namely dropout. Additionally, the DenseNet offers several advantages that address the shortcomings of the aforementioned current approaches, as outlined in Table 3.

**Figure 16:**
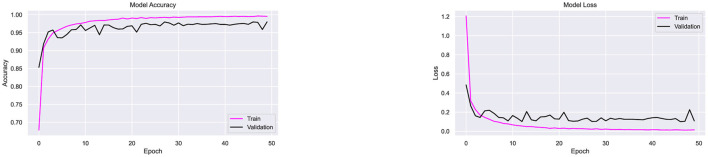
Performance testing of the CompoundDenseNet architecture on the Ekush dataset, showing both the training and validation accuracy and loss curves.

Since the proposed architecture has performed better with Adam optimizer, this combination is further tested on two more datasets, namely Ekush and CMATERdb. The image samples of 50 compound characters from Ekush are obtained, and these samples are divided into sets for training and validation purposes. Each class of 50 compound characters is represented by a training set consisting of 700 image samples and a validation set consisting of 175 samples. Subsequently, the data is inputted into the CompoundDenseNet model with a batch size of 20 and undergoes training for a total of 50 epochs. The recorded accuracy and loss for each epoch throughout the training and validation are presented in Figure 16.

The proposed architecture has shown superior performance on Ekush dataset as well. The approach demonstrated a remarkable overall classification accuracy of 98%, outperforming the performance of the state-of-the-art methods demonstrated in Table 10. The model achieved 99.56% training accuracy, with a training loss of 0.014 and validation loss of 0.1082 over 50 epochs. The stabilized validation loss within 50 epochs indicates that the model provides fast convergence and well generalization. The observation of a stabilized validation loss within 50 epochs implies the model exhibits fast convergence and effective generalization. Besides, the model has demonstrated superior accuracy on both the training data and the validation data, indicating a low risk of overfitting. The model have successfully categorized 8575 validation samples out of 8,750 validation samples. Figure 16 displays the accuracy and loss for each epoch during training and validation accordingly.

**Table 10:** Performance comparison of some existing studies on compound character recognition using the Ekush dataset.

Study references	Method	Accuracy (%)
Saha and Rahman (2024)	BanglaNet	97.32
Rakshit et al. (2023)	ResNet152V2	95.64
	Xception	95.58
Asraful et al. (2023)	CNN	97.54
Rabby et al. (2018b)	EkushNet	97.73
Shefin and Hashi (2021)	Multi–branch Deep CNN	95.32
Ahmed et al. (2023)	Deep convolutional neural network	95.30
Tani et al. (2023)	ResNet–50	97.92
This study	Proposed CompoundDenseNet	98.00

In Table 10, the methods used in some recent studies have been compared with our proposed CompoundDenseNet on the Ekush dataset. In study Rakshit et al. (2023), 11 CNN architectures have been tested on the Ekush dataset, and the results of the two best architectures are illustrated in Table 10. The primary cause stated in the study for the inaccurate prediction is the incorrect labeling of the images, which also serves as the primary cause of our study's classification loss. In order to prevent incorrect labeling, tedious and lengthy cleaning preprocessing is necessary. However, it is irrational to foresee a complete rectification of the dataset. A limitation of this research is that it explicitly presents the classification accuracy for individual numerals, basic characters, and the entire dataset. Compound characters are not considered separately. The proposed architecture of our study outperformed both the accuracies of basic characters and the entire dataset. Furthermore, our research has assessed the performance by determining the F1-Score, precision, and recall, all of which are absent in Rakshit et al. (2023). Study Asraful et al. (2023) has proposed a CNN-based approach, which has been tested on the Ekush dataset as well as the BanglaLekha-Isolated dataset. However, they evaluated the efficacy of their model using compound characters from the BanglaLekha-Isolated dataset but not the Ekush dataset without providing an explanation. Therefore, the accuracy achieved in this study for basic characters and digits of the Ekush dataset is mentioned in the table, which our proposed model has outperformed. Another constraint of this research is that while the formulas for calculating precision, recall, and F1-Score are presented, the corresponding results are not mentioned in the study. A multilayer CNN-based architecture is proposed in study Rabby et al. (2018b), which provides 97.73% classification accuracy to recognize the handwritten compound characters of the Ekush dataset. The study Rabby et al. (2018b) and Shefin and Hashi (2021) have used data augmentation techniques in the data preparation phase, which have not been used in our proposed study. Our model still achieves a higher level of accuracy than the models proposed in Rabby et al. (2018b) and Shefin and Hashi (2021). Besides, the compound characters that are mostly responsible for classification error in study Rabby et al. (2018b) are “ল্প” and “দ্দ”, which our proposed model has recognized with high accuracy. The study Tani et al. (2023) identified outliers as the initial cause of lower classification accuracy; however, those outliers were subsequently eliminated, and their proposed model attained 97.92% accuracy, which our model also has exceeded. In the final analysis, it can be claimed that our accuracy achieved using the Ekush dataset beat the results of all the contemporary methodologies.

**Figure 17:**
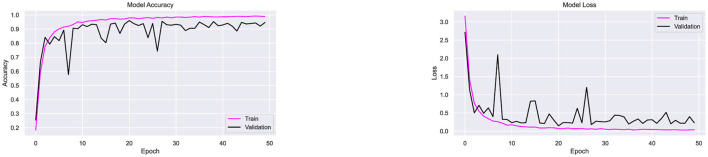
Performance testing of the CompoundDenseNet architecture on the CMATERdb dataset, showing both the training and validation accuracy and loss curves.

Following the completion of the experiment conducted on the Ekush dataset, the performance of the CompoundDenseNet model in conjunction with the Adam optimizer has been assessed using the CMATERdb dataset. In order to facilitate comparative analysis, 50 compound characters from the CMATERdb dataset have been selected, particularly those that match the compound characters present in the Ekush dataset. During the data collection phase, it is observed that the CMATERdb dataset has a much lower number of data instances per class in comparison to the other two datasets. Consequently, a reduced batch size of 5 is employed during the training process of the proposed model, spanning a total of 50 epochs. The proposed model has achieved 94.7% for classifying the compound characters of the CMATERdb dataset. The model has classified 1,190 compound samples correctly out of 1,257. The model's classification accuracy on the raw data of CMATERDb is notably poor. Additionally, an overfitting issue has been noted in Figure 17. This is evident from the model's high accuracy on the training data, whereas low accuracy on the validation data.

**Figure 18:**
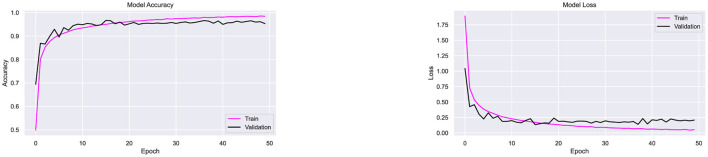
Performance testing of the CompoundDenseNet architecture on the augmented CMATERdb dataset, showing both the training and validation accuracy and loss curves.

Since the CMATERdb dataset has a limited number of samples per class (100–150), data augmentation techniques, including random rotation, color transformation, and shearing, were applied to improve the dataset's diversity and address class imbalance. These techniques helped enhance the model's generalization and reduced overfitting, resulting in a 1.5% improvement in validation accuracy. The CompoundDenseNet's overall classification accuracy on augmented data is 96.2%. Figure 18 demonstrates that data augmentation also significantly reduced data overfitting. This time, the model performed well on both the training and validation datasets, correctly classifying 1,208 out of 1,256 samples of compound characters. During the analysis of the classification report, it has become apparent that the model accurately classified all the samples of compound characters “ক্ষ”, “ক্ত”, “স্ক”, “ব্দ”, “ষ্ঠ”, “স্ম”, “জ্ব”, “ষ্ণ”, “দ্ভ”, “ঘ্ন”. On the other hand, the model has shown the least performance when classifying the compound character “জ্ঞ”, 5 instances of “জ্ঞ” wrongly classified as “ড্ড”. One of the primary constraints of the dataset involves its limited number of available data and the limited variety within it. The performance of the model on the CMATERdb dataset might potentially be enhanced by increasing both the variety and the amount of data.

This study also compares the performance of the proposed CompoundDenseNet with the recent available study on compound character classification using the CMATERdb dataset. The comparison has been shown in Table 11. The CompoundDenseNet outperforms the existing works tested on the CMATTERdb dataset. Study Jadhav et al. (2022) and Rabby et al. (2020) have utilized CMATERdb as their dataset but obtained a significantly lower degree of accuracy in compound character recognition compared to our research by a wide margin. In contrast, the study conducted by Chakraborty et al. (2022) has shown a notable level of accuracy using data augmentation, close to our own study. However, their suggested model exhibited a training accuracy of 81.75%, which is considerably lower than the corresponding validation accuracy. This discrepancy suggests a significant degree of underfitting in their model. This implies that the approach being presented exhibits a significant degree of bias and a relatively low level of variation. Several CNN-based models have been tested in study Islam M. S. et al. (2022) and achieved good accuracy. Similarly, some more CNN-based models have been evaluated using the CMATERdb dataset in study Ghosh et al. (2021), and our proposed model has outperformed all of these methods mentioned in Table 11. However, the analysis of the performance would have been more comprehensive if the training and validation graphs in study Islam M. S. et al. (2022) and Ghosh et al. (2021) had been illustrated.

**Table 11:** Performance comparison of some existing studies on compound character recognition using the CMATERdb dataset.

Study references	Method	Accuracy (%)
Jadhav et al. (2022)	CNN	87.00
Chakraborty et al. (2022)	DCNN	93.43
	DCNN with augmentation	96.17
Islam M. S. et al. (2022)	LeNet–5	91.80
	VGG–16	93.10
	ResNet–50	92.28
	RATNet–No–SAM–RB	95.75
Rabby et al. (2020)	Borno	86.09
Ghosh et al. (2021)	VGG16	90.70
	VGG19	92.07
	EfficientNetB3	92.80
	ResNet50V2	93.80
	ResNet50	94.91
	MobileNetV2	95.56
	NASNet	95.85
This study	CompoundDenseNet	96.20

Overall, our proposed CompoundDenseNet outperforms the accuracy of all previously reported supervised learning methods listed in Tables 9, 10. Notably, it achieves higher accuracy than the most recent study to our knowledge, which utilized BanglaNet—a hybrid of Inception, ResNet, and DenseNet architectures—for Bangla handwritten character recognition using the Ekush and BanglaLekha-Isolated datasets. This demonstrates that our model achieves state-of-the-art accuracy by leveraging these two datasets, which are among the largest and most diverse Bangla handwriting collections available.

## 5 Conclusion and future work

This study presents a DenseNet-based deep learning architecture, CompoundDenseNet, for recognizing Bangla handwritten compound characters, achieving remarkable accuracy across three key datasets: BanglaLekha-Isolated, Ekush, and CMATERdb. The BanglaLekha-Isolated dataset was prioritized due to its lower classification accuracy for compound characters compared to other datasets. Through comprehensive experiments, the Adam optimizer proved most effective with the DenseNet architecture, significantly enhancing performance across all datasets. The model achieved exceptional accuracies of 98.5%, 98%, and 96.2% on BanglaLekha-Isolated, Ekush, and CMATERdb respectively, outperforming prior state-of-the-art methods. The results highlight DenseNet's ability to extract complex features with minimal parameters and training time, as reflected in the confusion matrix analysis, which demonstrated robust performance despite variations in authorship and handwriting styles. However, the study also reveals areas for improvement. Misclassification rates are primarily caused by dataset noise, such as overwriting and unnecessary strokes, structural similarities among compound characters, and outliers. These issues can be mitigated through automated preprocessing techniques, including image denoising and anomaly-detection algorithms, to reduce manual effort. While CompoundDenseNet demonstrates strong performance, its limitations include high memory usage and sensitivity to hyperparameters like growth rate and dense block count, requiring iterative tuning. Future work could explore advanced architectures such as Vision Transformer or EfficientNet to further enhance accuracy in complex scenarios. Additionally, testing on more diverse datasets and analyzing gender- and age-based handwriting variations could improve robustness. Statistical significance tests could validate the results, paving the way for broader applications in Bangla handwriting recognition, including large-scale digitization of historic manuscripts, archival materials, and government records, especially in multicultural South Asian contexts, thereby advancing both scientific research and real-world implementations. Furthermore, while this study focuses on model performance, future research could investigate the integration of informative missingness in semi-supervised learning. This approach could improve the model's ability to handle missing strokes, partial characters, and ambiguous inputs, providing more robust performance in real-world OCR applications. Future work could also expand the dataset to include a broader set of compound characters to improve generalization and address the limitations caused by the current, limited set of characters in the study.

## Data Availability

The original contributions presented in the study are included in the article/supplementary material, further inquiries can be directed to the corresponding authors.
